# Functional impact of androgen‐targeted therapy on patients with castration‐resistant prostate cancer

**DOI:** 10.1002/bco2.179

**Published:** 2022-08-24

**Authors:** Tomasz M. Beer, Neal Shore, Alicia Morgans, Kerri Winters‐Stone, Jeffrey S. Wefel, Daniel J. George

**Affiliations:** ^1^ Oregon Health and Science University Knight Cancer Institute Portland Oregon USA; ^2^ Carolina Urologic Research Center Myrtle Beach South Carolina USA; ^3^ Dana‐Farber Cancer Institute Boston Massachusetts USA; ^4^ The University of Texas MD Anderson Cancer Center Houston Texas USA; ^5^ Duke Cancer Institute Durham North Carolina USA

**Keywords:** androgen deprivation therapy, androgen‐targeted therapy, castration‐resistant prostate cancer, cognitive function, physical function

## Abstract

**Context:**

Second‐generation androgen receptor inhibitors (ARIs) extend metastasis‐free survival, prolong overall survival, and delay symptoms when added to androgen deprivation therapy for the treatment of castration‐sensitive or castration‐resistant prostate cancer (CRPC). However, ARIs may adversely impact physical and cognitive function, thereby decreasing quality of life and prognosis.

**Objective:**

To evaluate the evidence regarding the potential effects of ARIs on physical and cognitive function and to contextualize how drug‐related adverse effects may influence treatment decisions in CRPC.

**Evidence acquisition:**

We performed a literature search using MEDLINE from January 1998 to June 2020 using terms relating to prostate cancer, androgen deprivation, and physical and cognitive function. We selected 61 publications for analysis.

**Evidence synthesis:**

Treatment‐induced deterioration in physical and cognitive function may impair the independence and well‐being of patients with CRPC. Patient‐reported outcomes from clinical trials of ARIs provide quantitative evidence of their impact on these domains, which appears to vary between ARIs, reflecting the different adverse event profiles of these agents. Thus, the risk of physical or cognitive dysfunction may be managed or mitigated by appropriate selection of treatment options. Studies in patients with CRPC have assessed the cognitive effects of ARIs with validated instruments, whereas quantitative analysis of the impact on physical function has been limited.

**Conclusion:**

Several validated instruments utilized for the assessment of physical and cognitive function in clinical studies have been adapted for clinical practice; however, consensus on the standardization of these assessments is required. Future clinical studies employing validated tools may generate data on the impact of ARIs and guide treatment decisions for patients with CRPC.

**Patient summary:**

We review the hormonal therapies used to treat men with prostate cancer and the effects they have on physical and cognitive function. We discuss how to measure these effects and how this may assist when choosing treatment.

## 1 INTRODUCTION

Approximately 30% of patients with localized prostate cancer (PC) experience rising prostate‐specific antigen concentrations within 5 years after definitive, localized therapy with either radical prostatectomy or radiotherapy.^1^ For recurrent, localized, locally advanced, and metastatic PC, androgen deprivation therapy (ADT) is a standard of care. Androgen deprivation may be achieved with bilateral orchiectomy or luteinizing hormone–releasing hormone agonists and antagonists.^2^

Treatment with ADT is often accompanied by acute and long‐term adverse effects. The safety profile of ADT is well described and affects multiple organ systems, including musculoskeletal, genitourinary, endocrine, cardiac, and the central nervous system (CNS). ADT‐related adverse events (AEs) have been associated with diminished physical function, which may impact the ability to perform daily activities independently.^3^, ^4^ As chronic comorbidities requiring the use of long‐term concomitant medication often co‐exist in patients with PC, the detrimental effects of ADT on physical and cognitive function can be compounded by the interactions of ADT with concurrent medications.^3^, ^5^

Most patients eventually develop resistance to ADT and progress to castration‐resistant PC (CRPC), despite sustained castrate levels of serum testosterone.^6^ It is hypothesized that resistance is driven by aberrant re‐activation of androgen receptor (AR) signalling through point and missense mutations, overexpression and amplification of the AR and its co‐regulators, intratumoural androgen biosynthesis, androgen indifference, and other mechanisms.^7^, ^8^

Several androgen‐targeted therapies are now approved for the treatment of advanced PC. Abiraterone acetate (a 17‐α‐hydroxylase/C17,20‐lyase [CYP17] androgen synthesis inhibitor) has been indicated for metastatic CRPC (mCRPC) and high‐risk metastatic castration‐sensitive PC (mCSPC) in combination with the glucocorticoid prednisone.^9^, ^10^, ^11^ The second‐generation androgen receptor inhibitors (ARIs) apalutamide and enzalutamide are approved for non‐metastatic CRPC (nmCRPC), mCRPC (enzalutamide only), and mCSPC based on their respective efficacy and safety results in phase 3 clinical trials.^12^, ^13^, ^14^, ^15^, ^16^ In 2019, darolutamide received approval for the treatment of nmCRPC.^17^ Darolutamide is a structurally distinct ARI with high molecular flexibility and polarity that may explain its low blood–brain barrier penetration.^18^

Clinical trials of second‐generation ARIs have demonstrated prolonged metastasis‐free survival and extended overall survival (OS) in nmCRPC when combined with ongoing ADT, but treatment with ARIs has been associated with a number of AEs that can adversely affect physical and cognitive function.^9^, ^12^, ^13^, ^17^, ^19^, ^20^ Interestingly, in a discrete choice experiment involving 149 US urologists and oncologists, survey participants were willing to trade substantial degrees of OS obtained with ARIs in patients with nmCRPC for a lower incidence of treatment‐related AEs of special interest, in particular, cognitive problems and fatigue, highlighting the importance of the benefit–risk profile in treatment selection decisions.^21^ Patients with nmCRPC and their caregivers also reported similar trade‐offs in terms of survival for lower risk and severity of AEs.^22^

This review aims to evaluate the evidence for the potential adverse effects of androgen‐targeted therapies on physical and cognitive function, and to consider how these drug‐related adverse effects and associated symptoms may influence treatment choices for patients with CRPC. We also discuss the need for prospective studies that objectively measure on‐treatment fluctuations in physical and cognitive function among men with CRPC using standardized metrics that may be adapted for use in clinical practice.

## 2 EVIDENCE ACQUISITION

We performed a literature search using MEDLINE and the following terms separately or in combination: prostate cancer, androgen deprivation therapy, body composition, muscle strength, lean body mass, physical capacity, physical impairment, physical function, daily living, fatigue, energy, vitality, cognitive decline, cognitive function, cognitive impairment, mental impairment, physical decline, physical dysfunction, physical performance, and comorbidities. Only English language articles were included, and the search was limited to those published between 1 January 1998 and 12 June 2020. Relevant papers were selected and reviewed based on their abstracts. After duplications were excluded, a total of 57 individual papers were included in this review. We subsequently included four additional papers of relevance that were published during the development of this manuscript. Studies involving patients with cancer types other than PC were included due to the paucity of available data in men with PC.

## 3 EVIDENCE SYNTHESIS

### 3.1 Multifactorial effects of worsening physical and cognitive function on daily life

**FIGURE 1:**
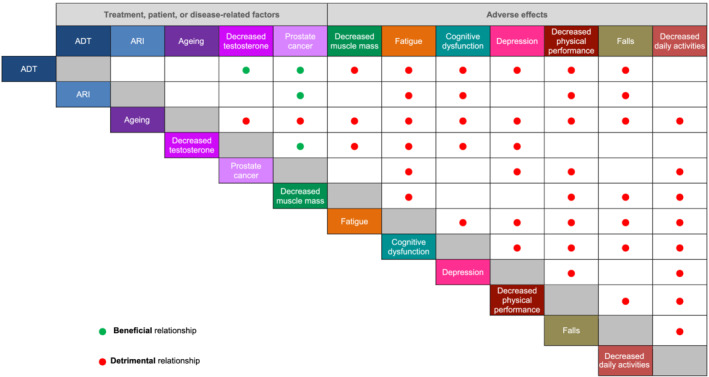
Multifactorial relationship between prostate cancer, treatments, ageing, and changes in physical function, cognition, and fatigue. Empty cells indicate no direct relationship relevant to this review reported. Some relationships are unidirectional (e.g., ADT and ARI have beneficial effects on cancer) whereas others are bidirectional (e.g., fatigue and depression).

In patients with cancer, a multifactorial relationship links changes in physical and cognitive function and fatigue to disease state, its treatments, and patient characteristics (Figure 1).^23^ Researchers have hypothesized that systemic anticancer therapies may accelerate cognitive and functional decline through cellular mechanisms of inflammation, which trigger neurotoxic cytokines, oxidative stress, DNA damage and/or faulty repair mechanisms, decreased telomere length, and cell senescence.^24^, ^25^, ^26^ Among cancer survivors, cognitive and physical deficits may become exacerbated by additional contributions from ageing, deconditioning, chronic comorbidities, concomitant medications, and genetic predisposition.^24^, ^27^

#### 3.1.1 Physical function

Physical function is the ability to engage in activities that require coordinated physical movements, ranging from self‐care to more complex activities that require a combination of skills, some of which are defined within a social context. Physical function is a multidimensional concept with four related subdomains: mobility (lower extremity function), dexterity (upper extremity function), axial ability (neck and back function), and the ability to perform instrumental activities of daily living (IADLs). Physical performance tests can objectively measure physical function and can detect decrements before they are self‐reported by patients.^28^

In contrast, physical activity is any bodily movement produced by skeletal muscles that requires energy expenditure and encompasses all forms of activity, including walking, gardening, work‐related activity, and active recreation, such as running, cycling, and other sports. In large observational studies, physical activity is commonly measured by self‐report. However, wearable activity monitors are increasingly being used to objectively assess the intensity, frequency, and duration of physical activity.^28^

Muscle weakness, loss of lean body mass, and weight gain are ADT‐associated treatment‐emergent AEs (TEAEs) that may contribute to deteriorating physical function.^4^ Patients with CRPC may be at an increased risk of falls, which may be compounded by age‐related declines in balance and mobility.^4^, ^29^ A cross‐sectional study of 280 men diagnosed with PC found that in comparison with patients who had never received ADT, current or past use of ADT predisposed men to twice as many falls (*p* = 0.002), four times the rate of recurrent falls (*p* < 0.001), and more fall‐related injuries (*p* = 0.01).^30^

#### 3.1.2 Fatigue

The central and/or peripheral mechanisms of cancer‐related fatigue (CrF) continue to elude confirmation, despite its high prevalence. A multifactorial pathogenesis of CrF has been posited to involve the interaction of cognitive, emotional, psychosocial, and somatic factors, with a highly variable clinical expression.^31^ An American Society of Clinical Oncology clinical practice guideline adaptation defined CrF as “a distressing, persistent, subjective sense of physical, emotional, and/or cognitive tiredness or exhaustion related to cancer or cancer treatment that is not proportional to recent activity and interferes with usual functioning.”^32^ Fatigue may persist for several months or years after therapy. Patients experiencing fatigue have reported impaired ability to exercise, participate in productive employment, socialize, and perform IADLs.^31^

#### 3.1.3 Cognitive function

The myriad symptoms of cognitive impairment manifest as deficits in attention, memory, executive function, visuospatial ability, and processing speed,^33^, ^34^ which may contribute to slowed reactions and diminished physical function.

The evidence suggesting a link between ADT and treatment‐emergent cognitive impairment in men with PC remains controversial.^35^, ^36^, ^37^, ^38^ A meta‐analysis of 14 studies, comprising a total of 417 patients with PC who received ADT, detected significantly worse performance on visuomotor tasks, such as the Block Design test, paper folding, and the Rey‐Osterrieth Complex Figure test (*g* = −0.67; *p* = 0.008) than untreated patients or controls.^35^ Studies with shorter times to follow‐up reported a greater magnitude of visuomotor deficit (*p* = 0.04).^35^ Similarly, overt cognitive dysfunction^39^ or structural and functional brain disturbances^40^, ^41^ have been reported within 6 months of starting ADT, whereas no long‐term deterioration in cognitive function was found.^37^ These findings suggest that the deleterious effects of ADT occur early in the treatment course and may dissipate over time.

In 2015, Gonzalez and colleagues reported a higher risk of cognitive impairment among patients with PC who received ADT at 6 and 12 months after initiation of treatment than age‐ and education‐matched controls in a prospective trial designed to assess impact of treatment on cognition (*p* < 0.05; *N* = 230; men treated with prostatectomy, *n* = 84; healthy men, *n* = 88; men treated with ADT, *n* = 58).^39^

Alibhai and colleagues found no consistent evidence of adverse cognitive effects from up to 36 months of continuous ADT. Their prospective, matched cohort study evaluated 87 patients treated with ADT for non‐metastatic PC (nmPC), 86 ADT non‐users with nmPC, and 86 healthy controls. The effect of ADT on cognitive function was assessed at 6 and 12 months from the time of treatment initiation with an extensive neuropsychological battery of 14 cognitive tests comprising eight cognitive domains. Effect sizes were negligible to small (range, −0.28 to 0.03) for all tests of attention, verbal/visual learning and memory, processing speed, and executive functions of working memory and cognitive flexibility.^36^ Fifty‐two patients from the original ADT cohort (67.5%) were followed for an additional 2 years, and median on‐treatment time from study start was 29.3 months. When compared with both the ADT non‐users and healthy controls, no differences in cognitive scores from baseline to 36 months were identified among ADT users for 13 of the 14 tests of cognitive function.^37^

In a prospective study of event‐based (EBPM) and time‐based (TBPM) prospective memory in 118 men, Yang and colleagues administered neuropsychological tests to 43 patients treated with ADT for PC, 35 patients with PC who did not receive ADT, and 40 healthy controls matched for age and education.^38^ Investigators found the ADT group scored lower than both the non‐ADT group and the controls on EBPM tasks, whereas no significant difference in the performance of TBPM tasks was detected among the three groups. In addition, ADT users demonstrated significantly lower scores in attention, memory, and information processing speed when compared with the other two groups, suggesting that a selective reduction in EBPM may occur in men treated with ADT that is potentially associated with a change in function and structure of the prefrontal cortex induced by androgen deprivation.^38^

These findings were supported by Chao and colleagues in their structural magnetic resonance imaging (MRI) study that evaluated comparative changes in cerebral grey matter among patients with PC who received (*n* = 12) or did not receive (*n* = 12) ADT. On MRI, decreased grey matter volumes in frontal and prefrontal cortical structures were detected among the 12 patients undergoing ADT. The reduced grey matter volume of the primary motor cortex correlated with an increased response time, implying a processing speed inefficiency for target detection during administration of the N‐back task.^41^ Results of this cerebral morphometry experiment corroborated those of a previous, prospective study by the same investigators concluding that ADT induced an altered activation of the medial prefrontal cortex on functional MRI during the stop‐signal task (cognitive control) in the active treatment cohort of patients with nmPC (*n* = 15).^40^

### 3.2 Strategies for mitigating decline in physical and cognitive function of patients with PC

#### 3.2.1 Treatment decision‐making

While most factors affecting the physical or cognitive function of patients with PC cannot be mitigated, the potential for anticancer therapy‐related AEs should be considered when treatment options are discussed. For example, according to a meta‐analysis of phase 3 studies, the risk of falls, fractures, dizziness, mental impairment, and fatigue can be significantly reduced with use of darolutamide versus enzalutamide or apalutamide.^42^ In the real‐world observational AQUARiUS and REAAcT studies, enzalutamide was associated with more cognitive dysfunction^43^ and fatigue^43^, ^44^ than was abiraterone acetate. However, abiraterone acetate is associated with higher rates of cardiovascular disease, hepatic dysfunction, and acute kidney injury, and related hospitalizations, than is enzalutamide.^45^, ^46^, ^47^, ^48^, ^49^ In addition, abiraterone acetate can induce mineralocorticoid‐related electrolyte imbalances,^11^ necessitating cotreatment with a corticosteroid (typically prednisone), which can lead to increased fracture risk and neuropsychiatric disturbances, depending on dose and duration of treatment.^50^ Some methods of improving tolerability by modifying the dose, such as starting at a low dose or, in the case of abiraterone acetate, taking treatment with food, have also been tried.^51^, ^52^, ^53^, ^54^, ^55^, ^56^, ^57^ These approaches, however, alter drug exposure and, particularly in the case of a food effect on abiraterone exposure, the impact on treatment effectiveness and safety is inconsistent; as a result, the prescribing information for abiraterone acetate mandates against taking the drug with food.^58^, ^59^ The need for dose reduction may be higher in patients receiving enzalutamide than in those receiving abiraterone acetate.^60^, ^61^

Despite the inherent advantages of an interactive relationship between patient and clinician, evidence suggests that healthcare professionals may underestimate the impact and severity of TEAEs, and studies have noted disparities between healthcare provider and patient assessments of ADT‐induced physical, sexual, urinary, and bowel function, fatigue, and bone pain.^62^ Effective communication between patients and their healthcare providers is essential to devising an appropriate individualized treatment plan. Additional strategies, as outlined below, may further help to maintain the independence, physical function, and cognitive abilities of patients treated for CRPC.

#### 3.2.2 Physical strategies

A growing body of evidence suggests that participation in exercise programmes can attenuate the loss of physical functioning and muscle mass. Appendicular or whole‐body skeletal muscle mass and physical performance can be assessed using the Short Physical Performance Battery (sPPB) and the Timed Up and Go (TUG) test, and by measuring gait speed, respectively.^63^ Exercise programmes have also demonstrated improvements in physical strength, endurance, and cardiorespiratory fitness of patients, and can ameliorate symptoms of fatigue resulting from androgen‐targeted therapy.^64^, ^65^

Recently published exercise guidelines for cancer survivors recommend that physical function can be improved with a minimum of 30 min of aerobic and/or resistance training at least three times per week for at least 8 to 12 weeks.^65^ Resistance training has been reported to increase muscle strength and physical function in men with PC and may be a particularly effective countermeasure to several ADT‐induced adverse effects. While patients with PC should strive to adopt an exercise regimen that incorporates elements of resistance training, aerobics, and balance training, clinicians can reassure their patients that any type, frequency, or duration of exercise at tolerable levels of intensity can have some benefit to overall health and well‐being.^66^, ^67^

#### 3.2.3 Cognitive function strategies

Cognitive rehabilitation can boost attention and memory in patients with cancer who experience treatment‐related cognitive decline. Group sessions that focus on psychoeducation and cognitive exercises to reinforce concentration and recall can alleviate perceived symptoms of cognitive impairment and improve objective measures of executive function.^68^

In older adults (≥60 years), physical activity is associated with a reduced risk of cognitive decline, dementia, and depression.^69^ In cancer survivors, hand grip strength, an indicator of muscle function, is associated with better cognitive function.^70^ Physical exercise, especially supervised aerobic and resistance exercise for at least 12 weeks, can help to ameliorate anxiety and depressive symptoms and improve social and cognitive function.^65^, ^71^, ^72^

### 3.3 Assessing the impact of ARIs on daily living in recent CRPC trials

Optimal treatment of CRPC with novel androgen‐targeted therapies requires a balance between clinical benefits and the potential for TEAE impact on physical and cognitive function. Currently, real‐world experience with androgen‐targeted therapy and its associated risk of physical and cognitive deficits is lacking and information to guide treatment decisions is limited to safety data from clinical trials conducted in controlled patient populations and a few observational studies. While phase 3 clinical trials for enzalutamide (PROSPER, PREVAIL), apalutamide (SPARTAN), darolutamide (ARAMIS), and abiraterone acetate (COU‐AA‐301 and COU‐AA‐302) incorporated a range of quality‐of‐life measures, specific evaluation of cognitive and physical function was not undertaken (Tables S1 and S2). With respect to drug safety, the combination of these androgen‐targeted therapies with ADT has been associated with higher rates of TEAEs that might impact physical and cognitive function when compared with ADT alone. These include weight loss, decreased appetite, fatigue, asthenia, falls, dizziness, mental impairment disorders, arthralgia, and fractures (Table S1).^10^, ^12^, ^13^, ^14^, ^17^, ^43^, ^44^, ^73^

Cognitive function and fatigue were specifically investigated in head‐to‐head comparisons of enzalutamide and abiraterone acetate in patients with mCRPC in the observational REAAcT and AQUARiUS studies (Table S1).^43^, ^44^ In both studies, clinically meaningful impairment in cognition and fatigue was reported more frequently with enzalutamide than abiraterone acetate. Although no between‐group differences were reported for scores on tools evaluating cognitive function (Cogstate tests, Functional Assessment of Cancer Therapy‐Cognitive Function [FACT‐Cog]) in REAAcT, a number of cognition‐related items from patient‐reported outcome (PRO) measures (FACT‐Cog, European Organisation for Research and Treatment of Cancer Quality of Life Questionnaire) favoured abiraterone acetate over enzalutamide. Notably more fatigue and greater deterioration on the Functional Assessment of Chronic Illness Therapy‐Fatigue scale was reported with enzalutamide than abiraterone acetate.^44^ Similarly, in AQUARiUS, men treated with abiraterone acetate were significantly less likely to experience exacerbations of usual fatigue or fatigue interference than those treated with enzalutamide.^43^

A meta‐analysis of neuropsychiatric AEs observed in the randomized clinical trials of enzalutamide or abiraterone acetate for the treatment of CRPC remarked on the evident increased risk of headache, insomnia, dizziness, and restless leg syndrome with enzalutamide versus placebo, while no such CNS associations were identified for abiraterone acetate. Notably, patients randomized to either agent experienced a significantly higher risk of falls compared with patients assigned to placebo.^74^

Data from the pivotal phase 3 clinical studies for enzalutamide, apalutamide, and darolutamide have demonstrated that when used in combination with ADT, these second‐generation ARIs are associated with TEAEs that may impact physical and cognitive function (Table S1). Specifically, in the primary analysis of the PROSPER study (median follow‐up: 23.0 months), fatigue, falls, and fractures occurred more commonly in patients who received enzalutamide plus ADT than those who received placebo plus ADT.^13^ The incidence of these AEs was also increased with enzalutamide in the final analysis of PROSPER (median follow‐up: 48.0 months), with the addition of cognitive and memory impairment, which included disturbances in attention, cognitive disorders, amnesia, Alzheimer's disease, dementia, senile dementia, mental impairment, and vascular dementia.^19^ In the primary analysis of the SPARTAN study (median follow‐up: 20.3 months), apalutamide and ADT treatment was associated with an increased incidence of fatigue, arthralgia, weight loss, falls, and fracture when compared with placebo and ADT.^12^ Increased incidences of fatigue, arthralgia, weight loss, and falls were also reported with apalutamide versus placebo in the final analysis of SPARTAN (median follow‐up: 52.0 months).^20^ Lastly, in the primary analysis of the ARAMIS study (median follow‐up: 17.9 months), the incidence of falls, fractures, and mental impairment was comparable between patients who received darolutamide and placebo, both administered in combination with ADT, while darolutamide plus ADT was associated with an increased incidence of fatigue.^17^ In the final analysis of ARAMIS (median follow‐up: 29.0 months), the incidence of falls, fractures, mental impairment disorders, and fatigue at the end of the double‐blind period remained consistent with that observed in the primary analysis.^9^

When analysing the AE profiles of the second‐generation ARIs in nmCRPC, it is important to appreciate that absolute risk of AEs in the placebo arms varied across the pivotal trials. A pooled analysis comparing the absolute risk in the SPARTAN placebo arm (given as 1.0) against those of ARAMIS and PROSPER demonstrated that the absolute risk of AEs was 0.46‐fold and 0.56‐fold lower, respectively. However, this analysis also recognized the substantial heterogeneity in collection of AEs, data reporting, interpretative practices, and patient experiences across seven randomized, placebo‐controlled trials with enzalutamide, apalutamide, and darolutamide in a total of 9215 patients with either mCRPC, nmCRPC, or mCSPC. The authors stressed that in the absence of inferential statistics, it is not possible to draw rigorous conclusions about relative drug safety between treatment and placebo arms, emphasizing the need for improved quantification of AE profiles.^75^ In a meta‐analysis of currently available ARIs, darolutamide showed significantly lower rates of falls, fractures, and rash versus apalutamide and significantly lower rates of falls, dizziness, mental impairment, fatigue, and severe fatigue versus enzalutamide.^42^

Several additional trials of androgen‐targeted therapies have adopted PROs to assess treatment‐related fatigue and objective measures to assess cognitive function in patients with CRPC (Table S1). These studies have been designed to reinforce available information on how these therapies impact daily life and the factors that influence patient preference when initiating treatment for CRPC. Currently, no studies in patients with CRPC objectively assess the effects of ARIs on physical function with validated instruments. Such quantifiable measures may convey less bias than questionnaires and may detect decremental changes in physical function earlier than self‐reports. Addressing this data gap may provide a better understanding of the impact of androgen‐targeted therapies on the ability to perform IADLs, and thus to tailor treatment decisions accordingly for patients with CRPC.

### 3.4 Objective measures of physical and cognitive function

A number of objective measures of physical and cognitive function have been used in several clinical studies (Table S3). The adoption of objective assessments in clinical practice is needed to ensure the recognition and optimal management of physical and cognitive impairment in routine care of patients with CRPC.

A vital component of managing cognitive impairment in older patients with cancer is the identification of pre‐existing cognitive changes prior to initiation of anticancer therapy. Brief, objective measures present the most feasible options in oncology clinical practice. Proactive assessment in the clinic may reveal the potential impact of disease or its treatment on the ability to independently execute critical life roles.^34^

Tools for routine objective assessment of physical or cognitive function by a medical oncologist in patients with PC have not been validated. General screening tools, such as the short G8 and Mini‐Cog tests, are recommended in European guidelines.^5^, ^76^, ^77^ Physicians need to understand the strengths and limitations of these screening tools, and combine this information with their knowledge of their individual patients, to identify patients in need of more comprehensive assessment, potentially with referral for specialized neuropsychological assessment.

## 4 CONCLUSIONS

All androgen‐targeted therapies can adversely affect the physical and cognitive status of patients with PC. Treatment‐induced deterioration in physical function and cognition may impact both the independence and the well‐being of patients with CRPC. The clinical benefit–risk profile of androgen‐targeted therapies is an especially important consideration in patients with nmCRPC, as they are largely asymptomatic from their malignancy and may anticipate an active lifestyle with longer life expectancy than those with mCRPC.

Effective management of older patients with CRPC requires pre‐treatment evaluation of their health status, ideally with a comprehensive geriatric assessment tool.^5^ It is essential that clinicians establish personalized management plans based on the pre‐treatment physical and cognitive function of their patients with CRPC, as this approach may minimize or attenuate the risk of adverse effects induced by androgen ablation. When adding androgen‐targeted therapy to ongoing ADT, shared decision‐making is framed by the determinants of age, the number and severity of non‐malignant comorbidities, the ability to self‐care and self‐administer medication, the drug safety profile, chronic concomitant medications, and drug–drug interactions. Resources such as plain language summaries (see Figure S1) are designed to inform patients of the factors affecting their care and may enable them to effectively participate in shared decision‐making conversations. Consideration of the impact of androgen‐targeted therapy on patients with PC should extend beyond potential AEs to their broader effects on cognitive function and general well‐being throughout the diagnostic and therapeutic journey.^5^, ^78^

While several validated instruments to assess the onset and extent of physical and cognitive impairment have been adapted to clinical practice, a consensus on uniform standards of evaluation has yet to be attained. Future clinical studies using validated measurements of physical and cognitive function may provide objective metrics on the impact of androgen‐targeted therapies on daily living and help guide the optimal treatment choice for patients with CRPC.

## CONFLICT OF INTEREST

Tomasz M. Beer has received research funding from Alliance Foundation Trials, Astellas Pharma, Bayer, Boehringer Ingelheim, Corcept Therapeutics, Endocyte Inc., Freenome, Grail Inc., Harpoon Therapeutics, Janssen Research & Development, Medivation, Inc., Sotio, Theraclone Sciences/OncoResponse, and Zenith Epigenetics. Tomasz M. Beer has received consulting fees from Arvinas, Astellas Pharma, AstraZeneca, Bayer, Bristol Myers Squibb (BMS), Constellation, Grail Inc., Janssen, Myovant Sciences, Pfizer, and Sanofi. Tomasz M. Beer has stock ownership in Arvinas Inc. and Salarius Pharmaceuticals. Neal Shore has received consulting fees from AbbVie, Amgen, Astellas, AstraZeneca, Bayer, BMS, Boston Scientific, Clovis Oncology, Cold Genesys, Dendreon, Exact Imaging, Exact Sciences, FerGene, Foundation Medicine, Genesis Care, Invitae, Janssen, MDxHealth, Merck, Myovant, Myriad, Nymox, Pacific Edge, Pfizer, Phosphorous, Propella, Sanofi Genzyme, Sesen Bio, Tolmar, and UroGen. Neal Shore has received speaker's bureau fees from Astellas, AstraZeneca, Bayer, Clovis Oncology, Janssen, Merck, Pfizer, and Guardant Health. Alicia Morgans: Bayer, Astellas, AstraZeneca, Clovis, Dendreon, AAA, Myovant, Blue Earth, Sanofi, Janssen, Seattle Genetics, and Pfizer. Kerri Winters‐Stones has no conflicts of interest to declare. Jeffrey S. Wefel has been a consultant or participated in an advisory board for AngioChem, Bayer, GT Medical Technology, Novocure, Roche, and Vanquish Oncology. Daniel J. George's conflicts of interest: American Association for Cancer Research, Astellas, AstraZeneca, Axess Oncology, Bayer HealthCare, BMS, Calithera, Capio Biosciences, Constellation Pharma, EMD Serono, Exelixis, Inc., Flatiron, Ipsen, Janssen Pharma, Merck Sharp & Dohme, Michael J. Hennessey Associates, Millennium Med Publishing, Modra Pharma, Myovant Sciences, Inc., NCI GU Steering committee member, Nektar Therapeutics, Novartis, Physician Education Resource, LLC, Pfizer, Propella Therapeutics, RevHealth, LLC, Sanofi, UroGPO, UroToday, and Vizuri Health Sciences, LLC.

## AUTHOR CONTRIBUTIONS

All authors contributed to the conception and scope of the review. All authors contributed to the writing and critical review of the article, with medical writing support from Scion and OPEN Health. All authors approved the final version for submission.

## Supporting information


**Figure S1.** Plain language summary infographic.
**Table S1.** Assessment of patient‐reported outcomes in recent clinical trials with CRPC patients.
**Table S2.** Domains of validated patient‐reported outcome assessment instruments.
**Table S3.** Assessment tools for physical and cognitive function in patients with cancer.
