# Supplementary material for: Functional impact of androgen‐targeted therapy on patients with castration‐resistant prostate cancer
Source: BJUI Compass. 2022 Aug 24;3(6):424–33. doi: 10.1002/bco2.179 (PMC9579880; doi:10.1002/bco2.179)
Supplement: Supplementary file 1 — Figure S1. Plain language summary infographic. Table S1. Assessment of patient‐reported outcomes in recent clinical trials with CRPC patients. Table S2. Domains of validated patient‐reported outcome assessment instruments. Table S3. Assessment tools for physical and cognitive function in patients with cancer. [file BCO2-3-424-s001.docx]

## Supplementary Appendix to:

Androgen-Targeted Therapy and Its Functional Impact on Patients With Castration-Resistant Prostate Cancer

Tomasz M. Beer^a^, Neal Shore^b^, Alicia Morgans^c^, Kerri Winters-Stone^a^, Jeffrey S. Wefel^d^, Daniel J. George^e^

^a^Oregon Health & Science University, Knight Cancer Institute, Portland, OR, USA ([Beert@ohsu.edu](mailto:Beert@ohsu.edu); [wintersk@ohsu.edu](mailto:wintersk@ohsu.edu)); ^b^Carolina Urologic Research Center, Myrtle Beach, SC, USA ([nshore@auclinics.com](mailto:nshore@auclinics.com)); ^c^Dana-Farber Cancer Institute, Boston, MA, USA ([aliciak_morgans@dfci.harvard.edu](mailto:aliciak_morgans@dfci.harvard.edu)); ^d^The University of Texas MD Anderson Cancer Center, Houston, TX, USA ([jwefel@mdanderson.org](mailto:jwefel@mdanderson.org)); ^e^Duke Cancer Institute, Durham, NC, USA ([daniel.george@duke.edu](mailto:daniel.george@duke.edu))

**Corresponding Author:**

Tomasz M. Beer, Oregon Health & Science University, Knight Cancer Institute Portland, OR, 97239 USA

Tel: 503-494-4393

Email: [Beert@ohsu.edu](mailto:Beert@ohsu.edu)

Supplemental Figure S1 – Plain language summary infographic


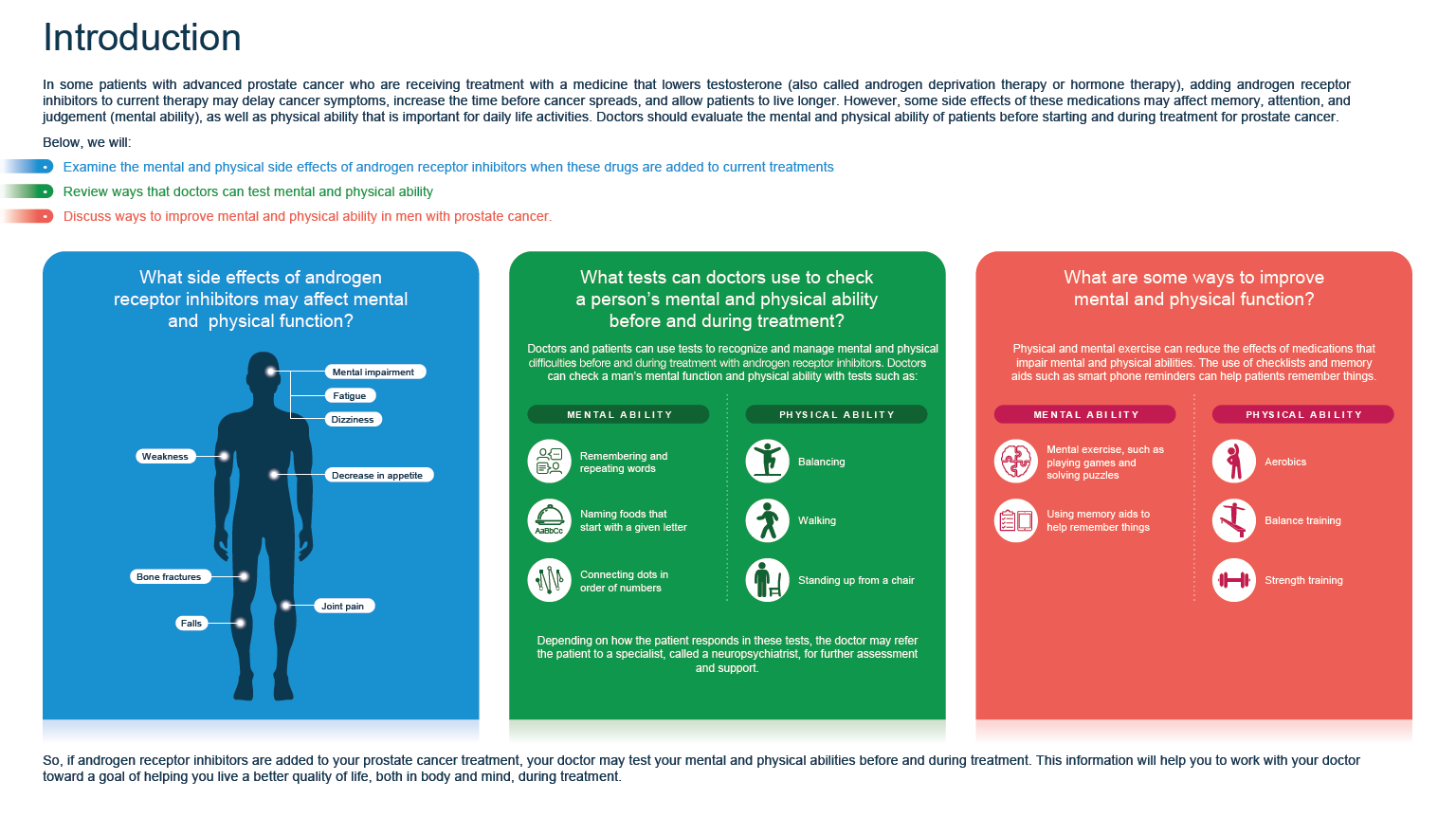


Supplemental Table S1 – Assessment of patient-reported outcomes in recent clinical trials with CRPC patients.

| **Trial** | **Description** | **Population** | **Primary and key secondary trial outcomes  (all drug vs PBO)** | **Assessment tools** | | | | **AEs*** | **Significant assessment outcomes (drug vs PBO)**^†^ |
| --- | --- | --- | --- | --- | --- | --- | --- | --- | --- |
|  |  |  |  | **Cognitive** | **QoL** | **Fatigue** | **Pain** |  |  |
| **Enzalutamide** | | | | | | | | |  |
| PROSPER ^1-3^  NCT02003924 | Randomized study to examine the safety and efficacy of enzalutamide compared with PBO in patients with nmCRPC  (N = 1401) | nmCRPC | MFS assessed from randomization until radiographic progression at any time, or death  36.6 vs 14.7 months  HR 0.29; 95% CI 0.24–0.35;  *p<*0.001  OS assessed from randomization to death from any cause  67.0 vs 56.3 months  HR 0.73; 95% CI 0.61–0.89;  *p*=0.001 | – | FACT-P  EQ-5D-5L  EQ-VAS  EORTC QLQ-PR25 | – | BPI-SF | Weight decrease  Decreased appetite  Fatigue  Asthenia  Fall  Dizziness  Mental impairment disorders | BPI-SF (pain severity): time to progression 36.83 months vs NR; HR 0.75; 95% CI 0.57–0.97; *p*=0.028  FACT-P total score: time to deterioration 22.11 vs 18.43 months; HR 0.83; 95% CI 0.69–0.99; *p*=0.037  EORTC QLQ-PR25: time to deterioration  Bowel symptoms and function: 33.15 vs 25.89 months; HR 0.72; 95% CI 0.59–0.89; *p*=0.0018  Hormonal treatment-related symptoms: 33.15 vs 36.83 months; HR 1.29; 95% CI 1.02–1.63; *p*=0.035  Urinary symptoms: 36.86 vs 25.86 months; HR 0.58; 95% CI 0.46–0.72; *p<*0.0001  EQ-VAS: time to deterioration 22.11 vs 14.75 months; HR 0.75; 95% CI 0.63–0.90; *p*=0.0013 |
| PREVAIL ^4-6^  NCT01212991 | Randomized study to examine efficacy of enzalutamide compared with PBO to prolong OS and PFS in patients with mCRPC (N = 1717) who progressed on ADT but did not receive chemotherapy | mCRPC | OS assessed from randomization to death due to any cause  32.4 vs 30.2 months  HR 0.71; 95% CI 0.60–0.84;  *p<*0.001  PFS assessed from randomization to the first evidence of radiographic disease progression or death due to any cause  NR vs 3.9 months  HR 0.19; 95% CI 0.15–0.23;  *p<*0.001 | – | EQ-5D-3L  EQ-VAS  FACT-P | – | BPI-SF | Fatigue  Arthralgia  Asthenia  Weight loss  Back pain  Decreased appetite  Fall | EQ-5D-3L: average decline −0.042 vs −0.070 points;  *p<*0.0001  EQ-VAS: average decline −1.3 vs −4.4 points; *p<*0.0001 |
| EFFECT  NCT03124615  Recruiting: 2022 | Open-label trial to study the effect of enzalutamide dose reduction in patients who have been treated with enzalutamide for ≤3 months who experience Grade 3 fatigue or cognition change  (estimated N = 47) | mPC or nmPC | The proportion of patients who have an improvement in cognition/fatigue symptoms, assessment 1-year  post-enrollment | Improvement in fatigue and cognition symptoms, based on improvement in questionnaire responses | | | | – | – |
| PREMISE ^7^  NCT02495974 | Observational study to assess effectiveness and outcomes associated with enzalutamide treatment in patients with mCRPC (N = 1732), including cohorts without prior chemotherapy or abiraterone therapy (Cohort 1, n = 1171) and with prior chemotherapy but without prior abiraterone (Cohort 2, n = 418)  N = 1732 | mCRPC | Time to treatment failure (TTF) defined as the time from initiation to discontinuation of enzalutamide for any reason (measured up to 18 months)  Median TTF: Cohort 1 12.9 months; Cohort 2 8.4 months  Patients with treatment failure: Cohort 1 650/1171 (58.9%) Cohort 2 311/418 (74.4%) | – | EQ-5D-5L  FACT-P | – | BPI-SF | Fatigue  Asthenia  Decreased appetite  Bone pain  Arthralgia | Mean scores at baseline, 9 months:  Cohort 1: EQ-VAS: 68.0, 71.9 FACT-P: 109.5, 113.9 BPI-SF severity: 2.01, 1.55 BPI-SF interference: 2.00, 1.57  Cohort 2: EQ-VAS: 65.2, 71.8 FACT-P: 107.0, 111.6 BPI-SF severity: 2.22, 1.89 BPI-SF interference: 2.56, 2.01 |
| **Enzalutamide and abiraterone acetate** | | | | | | | | |  |
| REEAcT ^8^  NCT02663193 | Multicenter, two-arm, open-label, prospective, observational study to characterize the tolerability profiles of treatment with enzalutamide or abiraterone acetate and QoL  N = 100  Timeframe: baseline and 2 months | mCRPC | Change from baseline in Cogstate tests, EORTC QLQ-C30, FACT-Cog, FACIT-Fatigue, and participant or caregiver observational questionnaire at Month 2 | Cogstate tests  FACT-Cog | EORTC QLQ-C30  Participant or caregiver observational questionnaire | FACIT-Fatigue | – | Fatigue | FACIT-Fatigue score: change from baseline  Enzalutamide group: –4 points; 95% CI −6.61 to −1.39  Abiraterone group: −0.01 points; 95% CI −2.40 to +2.38 |
| AQUARiUS ^9^  NCT02813408 | Prospective observational study to investigate the effect of abiraterone acetate and enzalutamide on HRQoL, fatigue, pain, cognitive function, and medical resource use  N = 211 (105 included in initial analysis)  Timeframe: 12 months | mCRPC | HRQoL (EORTC QLQ-C30), fatigue (BFI-SF), pain (BPI-SF), cognitive function (FACT-Cog) assessed over 12 months  Medical resource use, assessed at baseline until end of the follow-up period (up to 18 months) | FACT-Cog | EORTC QLQ-C30 | BFI-SF | BPI-SF | ^§^ | FACT-Cog: change from baseline (abiraterone acetate vs enzalutamide)  6.64 points; 95% CI 0.84–12.43;  *p*=0.025  BFI-SF usual level of fatigue: change from baseline (abiraterone acetate vs enzalutamide) −1.41 points; 95% CI −2.74 to −0.08; *p*=0.038  Fatigue interference: −1.20 points; 95% CI −2.31 to −0.08; *p*=0.036  EORTC QLQ-C30 cognitive function: change from baseline (abiraterone acetate vs enzalutamide) 11.82 points; 95% CI 0.84–22.79;  *p*=0.035 |
| **Apalutamide** | | | | | | | | |  |
| SPARTAN ^10-13^  NCT01946204 | Randomized phase 3 study to examine the safety and efficacy of apalutamide compared with PBO in patients with high-risk nmCRPC  (N = 1207) | nmCRPC | MFS assessed from randomization until radiographic progression at any time, or death  40.5 vs 16.2 months  HR 0.28; 95%CI 0.23–0.35;  *p<*0.001  OS assessed from randomization to death from any cause  73.9 vs 59.9 months  HR 0.78; 95% CI 0.64–0.96;  *p*=0.016 | – | FACT-P  EQ-5D-3L | – | – | Weight decrease  Arthralgia  Fatigue  Fracture  Fall  Dizziness | FACT-P  Difference in change in total score Cycle 21 *p*=0.0138 Cycle 25 *p*=0.0009 (1 cycle=28 days)  Median time to deterioration in total score 6.6 vs 8.4 mo; *p*=0.60 |
| **Abiraterone** | | | | | | | | |  |
| COU-AA-301 ^14, 15^  NCT00638690 | Randomized phase 3 study to compare the clinical benefit of abiraterone acetate plus prednisone with PBO plus prednisone for patients who have been treated with docetaxel (and possible additional chemotherapies)  N = 1195 | mCRPC | OS assessed from randomization to death of any cause  15.8 vs 11.2 months  HR 0.74; 95% CI 0.64–0.86;  *p<*0.0001 | – | FACT-P | – | BPI-SF | Fatigue  Arthralgia | Total FACT-P: time to deterioration 419 vs 253 days; HR 0.607; 95% CI 0.495–0.743; *p<*0.0001 |
| COU-AA-302 ^16, 17^  NCT00887198 | Randomized phase 3 study to compare the clinical benefit of abiraterone acetate plus prednisone with PBO plus prednisone in patients with mCRPC who had not received chemotherapy  N = 1088 | mCRPC | rPFS assessed from randomization to progression or death  16.5 vs 8.2 months  HR 0.52; 95% CI 0.45–0.61;  *p<*0.0001  OS assessed from randomization to death of any cause  34.7 vs 30.3 months; HR 0.81; 95% CI 0.70–0.93; *p=*0.0033 | – | FACT-P | – | BPI-SF | Fatigue  Arthralgia | BPI-SF (pain severity): time to progression 26.7 vs 18.4 months; HR 0.82; 95% CI 0.67–1.00; *p=*0.049  BPI-SF (pain interference): time to progression 10.3 vs 7.4 months; HR 0.79; 95% CI 0.67–0.93; *p=*0.005  Total FACT-P: time to deterioration 12.7 vs 8.3 months; HR 0.78; 95% CI 0.66–0.92; *p=*0.003 |
| **Darolutamide** | | | | | | | | |  |
| ARAMIS ^18-20^  NCT02200614 | Randomized phase 3 study to examine the safety and efficacy of darolutamide compared with PBO in patients with nmCRPC (N = 1509) | nmCRPC | MFS assessed from randomization until radiographic progression at any time, or death  40.4 vs 18.4 months;  HR 0.41; 95% CI 0.34–0.50;  *p<*0.001  OS assessed from randomization to death from any cause  NR vs NR HR 0.69; 95% CI 0.53–0.88;  *p=*0.003 | – | FACT-P  EQ-5D-3L  EORTC QLQ-PR25 | – | BPI-SF | Fatigue^║^ | BPI-SF: LSM, time-adjusted AUC, mean changes from baseline  Pain interference: 1.1 vs 1.3 points; difference −0.2; 95% CI −0.3 to −0.1  Pain severity 1.3 vs 1.4 points; difference −0.2; 95% CI −0.3 to −0.1  FACT-P total score: LSM, time-adjusted AUC, mean changes from baseline 112.9 vs 111.6 points; difference 1.3; 95% CI 0.4–2.1  FACT-P PCS: Median time to deterioration 11.1 vs 7.9 mo, HR 0.80, *p*=0.0005  EORTC QLQ-PR25: Median time to deterioration  Bowel symptoms 18.4 vs 11.5 mo, HR 0.78, *p*=0.0027  Hormonal treatment-related symptoms 18.9 vs 18.4 mo, HR 1.06, *p*=0.5237  Sexual activity 33.2 vs 30.1 mo, HR 0.82, *p*=0.0549  Sexual functioning 22.7 mo vs NR, HR 0.73, *p*=0.2815  Urinary symptoms 25.8 vs 14.8 mo, HR 0.64, *p*<0.0001 |
| ODENZA ^21, 22^  Recruiting:  2017–2020  NCT03314324 | Prospective, 24-week, randomized, open-label, multicenter, crossover phase 2 trial in patients with symptomatic or mildly symptomatic mCRPC (N = 249)  Randomized 1:1 to arm A = 12 weeks of enzalutamide followed by 12 weeks of darolutamide or to arm B = 12 weeks of darolutamide followed by 12 weeks of enzalutamide | mCRPC | Primary endpoint: patient preference between darolutamide and enzalutamide after completion of the second period of treatment (n = 200)  Darolutamide prefered: n = 97 (48.5%) Enzalutamide preferred: n = 80 (40.0%) No preference: n = 23 (11.5%) One-sided p=0.92  Most common factors influencing preference (darolutamide vs enzalutamide): Less fatigue 44% vs 29% Ease of taking the medication 37% vs 31% Better quality of life 36% vs 28% Ability to be more active 26% vs 15% Ability to concentrate 22% vs 15% Fewer falls 6% vs 3% | Cogstate | CES-D | BFI | – | Fatigue | Cogstate score: differences between darolutamide and enzalutamide:  Verbal learning (International Shopping List test): overall -0.54, p<0.0001  Verbal memory (International Shopping List test delayed recall) overall: -0.29, p=0.0075) |
| ARACOG ^23^  Recruiting  NCT04335682 | Randomized, 52-week phase 2 study to evaluate effects of darolutamide or enzalutamide on cognitive function in men with CRPC (N = 132) | nmCRPC or mCRPC | Primary endpoint: percent change in the maximally changed cognitive domain by  24 weeks in each study arm  Secondary endpoints: crossover from enzalutamide to darolutamide and darolutamide to enzalutamide, maximally changed cognitive domain, proportion of impaired patients, change in lowest ranking domain, improvements in cognitive function after crossover, PSA progression, PFS, PSA response rate | CANTAB  FACT-Cog | – | – | – | – | – |

*AEs that are likely to affect the physical activity of patients and occurred with a ≥2% incidence in patients in the treatment group compared with the placebo group (arthralgia, asthenia, bone pain, decreased appetite, dizziness, falls, fatigue, fractures, mental impairment disorders, pain, weight loss).

^†^Only significant results are reported in this table.

^§^None of the AEs reported met the inclusion criteria for this table.

^║^Grouped terms, for more information see publication ^18^.

ADT = androgen deprivation therapy; AE = adverse event; AUC = area under the curve; BFI = Brief Fatigue Inventory; BFI-SF = Brief Fatigue Inventory Short Form; BPI-SF = Brief Pain Inventory Short Form; CANTAB = Cambridge Neuropsychological Test Automated Battery; CES-D = Center for Epidemiologic Studies Depression Scale; C30 = 30-item core questionnaire; CI = confidence interval; COWA = Controlled Oral Word Association; CRPC = castration-resistant prostate cancer; CSPC = castration-sensitive prostate cancer; ECOG PS = Eastern Cooperative Oncology Group Performance Status; EORTC QLQ = European Organisation for the Research and Treatment of Cancer Quality of Life Questionnaire; EQ-5D-3L = EuroQoL 5-Dimensions 3-Levels health questionnaire; EQ-5D-5L = EuroQoL 5-Dimensions 5-Levels health questionnaire; EQ-VAS = EuroQoL 5-Dimensions 5-Levels health questionnaire visual analog scale; FACIT-Fatigue = Functional Assessment of Chronic Illness Therapy‑Fatigue; FACT-Cog = Functional Assessment of Cancer Therapy-Cognitive Function; FACT-P = Functional Assessment of Cancer Therapy-Prostate; HR = hazard ratio; HRQoL = health-related quality of life; HVLT-R = Hopkins Verbal Learning Test-Revised; LSM = least-squares mean; mCRPC = metastatic castration-resistant prostate cancer; MFS = metastasis-free survival; mPC = metastatic prostate cancer; nmCRPC = non-metastatic castration-resistant prostate cancer; nmPC = non-metastatic prostate cancer; NR = not reached; OS = overall survival; PBO = placebo; PFS = progression-free survival; PR25 = prostate cancer-specific 25-item questionnaire; PSA = prostate-specific antigen; QoL = quality of life; rPFS = radiographic progression-free survival; sPBB = short Physical Performance Battery; TMT = Trail Making Test; TUG = Timed Up and Go.

Supplemental Table S2 – Domains of validated patient-reported outcome assessment instruments.

|  | **BPI-SF ^24^** | **EORTC QLQ-PR25 ^25^** | **EORTC QLQ-C30 ^26^** | **EQ-5D-3L ^27^** | **EQ-VAS ^28^** |
| --- | --- | --- | --- | --- | --- |
| Domains | Pain severity  Pain interference  General activity  Mood  Walking ability  Work  Social relationships  Sleep  Enjoyment of life | Urinary symptoms  Incontinence aid  Bowel symptoms  Hormonal treatment-related symptoms | Physical function  Role function  Cognitive function  Emotional function  Social function  Pain  Fatigue  Nausea  Vomiting  Global health/QoL  Dyspnea  Appetite loss  Sleep disturbance  Constipation  Diarrhea | Mobility  Self-care  Usual activities  Pain/discomfort  Anxiety/depression  Three levels indicating  Level 1: No problem  Level 2: Some problems  Level 3: Extreme problems | Vertical visual analogue scale  Values between 100 = Best imaginable health and  0 = Worst imaginable health  Patients mark scale to assign a number to global assessment of their health |
|  | **FACT-P ^29^** | **FACT-Cog ^30, 31^** | **FACIT-Fatigue ^32-34^** | **EQ-5D-5L ^28^** | **BFI ^35^** |
| Domains | Physical wellbeing  Social/family wellbeing  Relationship with doctor  Emotional wellbeing  Functional wellbeing  Prostate cancer subscale: 12 items  Ranked on five-point Likert scale:  0–4 | Perceived cognitive impairments: 20 items  Comments from others:  4 items  Perceived cognitive abilities: 9 items  Impact on QoL: 4 items | 13-item scale  Ranked on five-point Likert scale:  0–4  Fatigued  Weak  Listless/washed out  Tired  Trouble starting things because I am tired  Trouble finishing things because I am tired  Energy  Everyday usual activities  In need of daytime sleep  Too tired to eat  Need help with usual activities  Frustrated by being too tired to do what I want to do  Must limit social activity because I am tired | Mobility  Self-care  Usual activities  Pain/discomfort  Anxiety/depression  5 levels indicating  Level 1: No problems  Level 2: Slight problems  Level 3: Moderate problems  Level 4: Severe problems  Level 5: Extreme problems | 0–10 rating scale  0 = No fatigue and  10 = As bad as you can imagine  Fatigue now  Usual level of fatigue:  During past 24 hours  Worst level of fatigue:  During past 24 hours  In past 24 hours how has fatigue interfered with:   - General activity - Mood - Walking ability - Normal work - Relations with others - Enjoyment of life |

BFI = Brief Fatigue Inventory; BPI-SF = Brief Pain Inventory-Short Form; C30 = 30-item core questionnaire; EORTC = European Organisation for Research and Treatment; EQ-5D-3L = EuroQoL 5-Dimensions 3-Levels; EQ-5D-5L = EuroQoL 5-Dimensions 5-Levels; EQ-VAS = EuroQoL Visual Analogue Scale; FACIT-Fatigue = Functional Assessment of Chronic Illness Therapy-Fatigue; FACT-Cog = Functional Assessment of Cancer Therapy-Cognitive Function; FACT-P = Functional Assessment of Cancer Therapy-Prostate; PR25 = prostate cancer-specific module; QLQ = quality of life questionnaire; QoL = quality of life.

Supplemental Table S3 – Assessment tools for physical and cognitive function in patients with cancer.

| Validated instrument | Characteristics tested | Advantages | Instrument properties | Assessment methods |
| --- | --- | --- | --- | --- |
| TUG ^36-38^ | Physical function:   - Balance - Functional mobility - Gait/ambulation - Risk of locomotive falls   Assistive devices permitted | - Valid - Reliable - Brief - Easy to administer | - Clinical performance-based measure of lower extremity function, mobility, and fall risk. - Construct validity: r = 0.75  (gait speed) - Thought to be sensitive to early changes in functional status   - Sensitivity: 87% (falls)   - Specificity: 87% (falls) | - Rise from chair, walk 3 meters (~10 feet) at usual gait speed, reverse course, return and resume seated position. Repeat once - Reference values (median time) for test completion by age:   - 60–69 years: 8.1 seconds   - 70–79 years: 9.2 seconds   - 80–99 years: 11.3 seconds - Shorter times indicate better performance. Slower times may indicate cognitive impairment - Assessment video: <https://vimeo.com/418985122/bdca024046> |
| sPPB ^39-41^ | Objective measure of balance, and functional capacity:   - Lower extremity physical performance status - Functional capability - Biological age   Assistive devices permitted | - Efficient - Practical - Brief | - Objective tool consisting of three different timed assessments: walking speed, chair stand, and balance time - Timed results are scored by predefined cut-off points - Performance scores range from 0 (worst) to 12 (best) - Internal consistency: 0.63–0.66   - Sensitivity (score <9): 0.54   - Specificity (score <9): 0.92 - Possibly predictive of death or nursing home placement | - Summary performance scale: 0–4 - Standing balance: each position sustained for 10 seconds   - Side-by-side: one foot alongside opposite foot   - Semi-tandem: heel against side of opposite big toe   - Full-tandem: heal in front of opposite toes - Walking speed: ambulate 8–10 feet at usual pace, repeat once - Chair stand: fold arms across chest and arise from straight-backed chair unassisted, repeat five times as quickly as possible |
| BFI ^35, 42, 43^ | - Fatigue severity - Correlates with measures of:   - Performance status   - Physiological markers of anemia and nutritional status - All known to be associated with fatigue | - Simple - Easy to understand | - Validated in patients with cancer - Construct validity:   - Interference: 0.597–0.861   - Severity: 0.806–0.872 - Internally stable (reliable):   - Internal consistency: 0.96   - Test-retest reliability: 0.90–0.93 | - Questionnaire based on the BPI - Nine items assess fatigue severity and interference with activities of daily living on a numeric scale from 1 to 10 at the time of the test and the past 24 hours   - 1 = “no fatigue/does not interfere”   - 10 = “fatigue as bad as you can imagine/completely interferes” |
| KPS ^44, 45^ | - Physical function of patients with cancer | - Decision aid for cancer management | - Although the KPS is widely used by clinicians, evidence suggests a variable inter-rater reliability as frequently determinations between HCPs are not comparable (~0.69) - Analyses have substantiated the predictive validity of the KPS for duration of survival when applied to a sample of terminal cancer patients | - A set of objective criteria to evaluate treatment efficacy - Based on tumor size, laboratory parameters (eg, anemia), remission length, and overall survival, measured using an 11-point scale correlating to percentage values describing three states:   - A (100–80%): able to carry on normal activity   - B (70–50%): unable to work, but able to live at home and self-care with varying assistance   - C (40–0%): unable to care for self; requires institutional/hospital care |
| HVLT-R ^46, 47^ | - Verbal episodic learning and memory - Six psychometrically equivalent forms available | - Valid - Reliable - Recommended by ICCTF | – | - Examiner-based evaluation - Normative data available to determine impairment relative to healthy population |
| TMT ^47-49^ | - Psychomotor speed, mental flexibility, and executive function | - Valid - Reliable - Recommended by ICCTF | – | - Examiner-based evaluation - Normative data available to determine impairment relative to healthy population |
| COWA ^47, 50^ | - Assesses phonemic fluency and lexical access - Two alternative forms available | - Valid - Reliable - Recommended by the ICCTF | – | - Examiner-based evaluation - Normative data available to determine impairment relative to healthy population |
| PHQ-9 ^51^ | - Major depressive disorder module of the full PHQ - Serves to provisionally diagnose depression and grade symptom severity in general medical and mental health settings - Final diagnosis requires a clinical interview and mental status examination to include the assessment of functional impairment and degree of distress | - Uni-dimensional questionnaire - Analyzes nine items including:   - Loss of interest   - Loss of energy   - Depression   - Sleep   - Concentration - A two-dimensional form has been used in patients with PC | - A two-dimensional form has been used in patients with PC | - Scored as 0 to 27 points - Results:   - 0–4: minimal or none   - 5–9: mild   - 10–14: moderate   - 15–19: moderately severe   - 20–27: severe - >4: consider a depressive disorder - >5: consider major depressive disorder |
| FACT-Cog ^52, 53^ | - Reliable and valid self-report measure of the nature and severity of cognitive impairments and their impact on quality of life in patients with cancer - Written to be behaviorally based whenever possible, rather than as a subjective impression of global function | - Employs both negative and positive wording - Specifically developed for use in focus groups of cancer patients and by clinical trial experts | - Has been validated in patients with cancer | - 50-item questionnaire - Comprised of 4 subscales:   - Perceived cognitive impairments (18 items)   - Perceived cognitive abilities  (7 items)   - Impact of perceived cognitive impairment on quality of life  (4 items)   - Comments from others on cognitive function (4 items) - Response scale is based on behavioral frequency, eg, one time per week - Possible scores: 0–28 |
| Accelerometry ^54^ | - Intensity, frequency, and duration of physical activity - Provides individual, objective, detailed and accurate information | - Small, lightweight device - Extensively validated | - Used in clinical studies | - Participant wears accelerometer for 7 days at each assessment time point - A recording of ≥10 hours of data is defined as a valid day - Records data for physical activity as counts per minute (cpm), with sedentary behavior defined as <100 cpm |

BFI = Brief Fatigue Inventory; BPI = Brief Pain Inventory; COWA = Controlled Oral Word Association; FACT-Cog = Functional Assessment of Cancer Therapy-Cognitive Function; HCP = healthcare professional; HVLT-R = Hopkins Verbal Learning Test, Revised; ICCTF = International Cognition and Cancer Task Force; KPS = Karnofsky Performance Scale; PC = prostate cancer; PHQ = Patient Health Questionnaire; QoL = quality of life; sPPB = Short Physical Performance Battery; TMT = Trail Making Test; TUG = Timed Up & Go.

# References

1. Hussain M, Fizazi K, Saad F, et al. Enzalutamide in men with nonmetastatic, castration-resistant prostate cancer. *N Engl J Med*. 2018; **378**(26):2465-74. <https://doi.org/10.1056/NEJMoa1800536>

2. Tombal B, Saad F, Penson D, et al. Patient-reported outcomes following enzalutamide or placebo in men with non-metastatic, castration-resistant prostate cancer (PROSPER): a multicentre, randomised, double-blind, phase 3 trial. *Lancet Oncol*. 2019; **20**(4):556-69. <https://doi.org/10.1016/s1470-2045(18)30898-2>

3. Sternberg CN, Fizazi K, Saad F, et al. Enzalutamide and survival in nonmetastatic, castration-resistant prostate cancer. *N Engl J Med*. 2020; **382**(23):2197-206. <https://doi.org/10.1056/NEJMoa2003892>

4. Beer TM, Armstrong AJ, Rathkopf DE, et al. Enzalutamide in metastatic prostate cancer before chemotherapy. *N Engl J Med*. 2014; **371**(5):424-33. <https://doi.org/10.1056/NEJMoa1405095>

5. Devlin N, Herdman M, Pavesi M, et al. Health-related quality of life effects of enzalutamide in patients with metastatic castration-resistant prostate cancer: an in-depth post hoc analysis of EQ-5D data from the PREVAIL trial. *Health Qual Life Outcomes*. 2017; **15**(1):130. <https://doi.org/10.1186/s12955-017-0704-y>

6. Loriot Y, Miller K, Sternberg CN, et al. Effect of enzalutamide on health-related quality of life, pain, and skeletal-related events in asymptomatic and minimally symptomatic, chemotherapy-naive patients with metastatic castration-resistant prostate cancer (PREVAIL): results from a randomised, phase 3 trial. *Lancet Oncol*. 2015; **16**(5):509-21. <https://doi.org/10.1016/s1470-2045(15)70113-0>

7. Payne H, Robinson A, Rappe B, et al. A European, prospective, observational study of enzalutamide in patients with metastatic castration-resistant prostate cancer: PREMISE. *Int J Cancer*. 2022; **150**(5):837-46. <https://doi.org/10.1002/ijc.33845>

8. Shore ND, Saltzstein D, Sieber P, et al. Results of a real-world study of enzalutamide and abiraterone acetate with prednisone tolerability (REAAcT). *Clin Genitourin Cancer*. 2019; **17**(6):457-63.e6. <https://doi.org/10.1016/j.clgc.2019.07.017>

9. Thiery-Vuillemin A, Poulsen MH, Lagneau E, et al. Impact of abiraterone acetate plus prednisone or enzalutamide on fatigue and cognition in patients with metastatic castration-resistant prostate cancer: initial results from the observational AQUARiUS study. *ESMO Open*. 2018; **3**(5):e000397. <https://doi.org/10.1136/esmoopen-2018-000397>

10. Smith MR, Saad F, Chowdhury S, et al. Apalutamide treatment and metastasis-free survival in prostate cancer. *N Engl J Med*. 2018; **378**(15):1408-18. <https://doi.org/10.1056/NEJMoa1715546>

11. Saad F, Cella D, Basch E, et al. Effect of apalutamide on health-related quality of life in patients with non-metastatic castration-resistant prostate cancer: an analysis of the SPARTAN randomised, placebo-controlled, phase 3 trial. *Lancet Oncol*. 2018; **19**(10):1404-16. <https://doi.org/10.1016/s1470-2045(18)30456-x>

12. Smith MR, Saad F, Chowdhury S, et al. Apalutamide and overall survival in prostate cancer. *Eur Urol*. 2021; **79**(1):150-8. <https://doi.org/10.1016/j.eururo.2020.08.011>

13. Oudard S, Hadaschik B, Saad F, et al. Health-related quality of life at the SPARTAN final analysis of apalutamide for nonmetastatic castration-resistant prostate cancer patients receiving androgen deprivation therapy. *Eur Urol Focus*. 2021:September 5, 2021 (epub ahead of print). <https://doi.org/10.1016/j.euf.2021.08.005>

14. Fizazi K, Scher HI, Molina A, et al. Abiraterone acetate for treatment of metastatic castration-resistant prostate cancer: final overall survival analysis of the COU-AA-301 randomised, double-blind, placebo-controlled phase 3 study. *Lancet Oncol*. 2012; **13**(10):983-92. <https://doi.org/10.1016/s1470-2045(12)70379-0>

15. Harland S, Staffurth J, Molina A, et al. Effect of abiraterone acetate treatment on the quality of life of patients with metastatic castration-resistant prostate cancer after failure of docetaxel chemotherapy. *Eur J Cancer*. 2013; **49**(17):3648-57. <https://doi.org/10.1016/j.ejca.2013.07.144>

16. Basch E, Autio K, Ryan CJ, et al. Abiraterone acetate plus prednisone versus prednisone alone in chemotherapy-naive men with metastatic castration-resistant prostate cancer: patient-reported outcome results of a randomised phase 3 trial. *Lancet Oncol*. 2013; **14**(12):1193-9. <https://doi.org/10.1016/s1470-2045(13)70424-8>

17. Ryan CJ, Smith MR, Fizazi K, et al. Abiraterone acetate plus prednisone versus placebo plus prednisone in chemotherapy-naive men with metastatic castration-resistant prostate cancer (COU-AA-302): final overall survival analysis of a randomised, double-blind, placebo-controlled phase 3 study. *Lancet Oncol*. 2015; **16**(2):152-60. <https://doi.org/10.1016/S1470-2045(14)71205-7>

18. Fizazi K, Shore N, Tammela TL, et al. Darolutamide in nonmetastatic, castration-resistant prostate cancer. *N Engl J Med*. 2019; **380**(13):1235-46. <https://doi.org/10.1056/NEJMoa1815671>

19. Fizazi K, Shore N, Tammela TL, et al. Nonmetastatic, castration-resistant prostate cancer and survival with darolutamide. *N Engl J Med*. 2020; **383**(11):1040-9. <https://doi.org/10.1056/NEJMoa2001342>

20. Smith MR, Shore N, Tammela TL, et al. Darolutamide and health-related quality of life in patients with non-metastatic castration-resistant prostate cancer: an analysis of the phase III ARAMIS trial. *Eur J Cancer*. 2021; **154**:138-46. <https://doi.org/10.1016/j.ejca.2021.06.010>

21. Colomba E, Jonas SF, Eymard J-C, et al. ODENZA: a French prospective, randomized, open-label, multicenter, cross-over phase II trial of preference between darolutamide and enzalutamide in men with asymptomatic or mildly symptomatic metastatic castrate-resistant prostate cancer (CRPC). *J Clin Oncol*. 2021; **39 (Suppl)**(15):5046. <https://doi.org/10.1200/JCO.2021.39.15_suppl.5046>

22. Colomba E, Jonas SF, Eymard JC, et al. 603P Objective computerized cognitive assessment in men with metastatic castrate-resistant prostate cancer (mCRPC) randomly receiving darolutamide or enzalutamide in the ODENZA trial. *Ann Oncol*. 2021; **32 (Suppl)**:S646-7. <https://doi.org/10.1016/annonc/annonc702>

23. ClinicalTrials.gov. Androgen receptor directed therapy on cognitive function in patients treated with darolutamide or enzalutamide (ARACOG) - NCT04335682. Retrieved from <https://clinicaltrials.gov/show/NCT04335682>. Accessed April 21, 2022

24. Cleeland CS, Ryan KM. Pain assessment: global use of the Brief Pain Inventory. *Ann Acad Med Singap*. 1994; **23**(2):129-38.

25. van Andel G, Bottomley A, Fosså SD, et al. An international field study of the EORTC QLQ-PR25: a questionnaire for assessing the health-related quality of life of patients with prostate cancer. *Eur J Cancer*. 2008; **44**(16):2418-24. <https://doi.org/10.1016/j.ejca.2008.07.030>

26. Aaronson NK, Ahmedzai S, Bergman B, et al. The European Organization for Research and Treatment of Cancer QLQ-C30: a quality-of-life instrument for use in international clinical trials in oncology. *J Natl Cancer Inst*. 1993; **85**(5):365-76. <https://doi.org/10.1093/jnci/85.5.365>

27. Rabin R, de Charro F. EQ-5D: a measure of health sta tus from the EuroQol Group. *Ann Med*. 2001; **33**(5):337-43. <https://doi.org/10.3109/07853890109002087>

28. Janssen MF, Pickard AS, Golicki D, et al. Measurement properties of the EQ-5D-5L compared to the EQ-5D-3L across eight patient groups: a multi-country study. *Qual Life Res*. 2013; **22**(7):1717-27. <https://doi.org/10.1007/s11136-012-0322-4>

29. Esper P, Mo F, Chodak G, Sinner M, Cella D, Pienta KJ. Measuring quality of life in men with prostate cancer using the Functional Assessment of Cancer Therapy-Prostate instrument. *Urology*. 1997; **50**(6):920-8. <https://doi.org/10.1016/s0090-4295(97)00459-7>

30. Wagner LI, Sweet J, Butt Z, Lai J-s, Cella D. Measuring patient self-reported cognitive function: development of the functional assessment of cancer therapy-cognitive function instrument. *J Support Oncol*. 2009; **7**(6):W32-9.

31. Costa DSJ, Loh V, Birney DP, et al. The structure of the FACT-Cog v3 in cancer patients, students, and older adults. *J Pain Symptom Manage*. 2018; **55**(4):1173-8. <https://doi.org/10.1016/j.jpainsymman.2017.12.486>

32. Yellen SB, Cella DF, Webster K, Blendowski C, Kaplan E. Measuring fatigue and other anemia-related symptoms with the Functional Assessment of Cancer Therapy (FACT) measurement system. *J Pain Symptom Manage*. 1997; **13**(2):63-74. <https://doi.org/10.1016/s0885-3924(96)00274-6>

33. Cella D, Eton DT, Lai JS, Peterman AH, Merkel DE. Combining anchor and distribution-based methods to derive minimal clinically important differences on the Functional Assessment of Cancer Therapy (FACT) anemia and fatigue scales. *J Pain Symptom Manage*. 2002; **24**(6):547-61. <https://doi.org/10.1016/s0885-3924(02)00529-8>

34. Bell ML, Dhillon HM, Bray VJ, Vardy JL. Important differences and meaningful changes for the Functional Assessment of Cancer Therapy-Cognitive Function (FACT-Cog). *J Patient Rep Outcomes*. 2018; **2**(48):1-11. <https://doi.org/10.1186/s41687-018-0071-4>

35. Mendoza TR, Wang XS, Cleeland CS, et al. The rapid assessment of fatigue severity in cancer patients: use of the Brief Fatigue Inventory. *Cancer*. 1999; **85**(5):1186-96. [https://doi.org/10.1002/(SICI)1097-0142(19990301)85:5<1186::AID-CNCR24>3.0.CO;2-N](https://doi.org/10.1002/(SICI)1097-0142(19990301)85:5%3c1186::AID-CNCR24%3e3.0.CO;2-N)

36. Steffen TM, Hacker TA, Mollinger L. Age- and gender-related test performance in community-dwelling elderly people: Six-Minute Walk Test, Berg Balance Scale, Timed Up & Go Test, and gait speeds. *Phys Ther*. 2002; **82**(2):128-37. <https://doi.org/10.1093/ptj/82.2.128>

37. Bohannon RW. Reference values for the timed up and go test: a descriptive meta-analysis. *J Geriatr Phys Ther*. 2006; **29**(2):64-8. <https://doi.org/10.1519/00139143-200608000-00004>

38. Herman T, Giladi N, Hausdorff JM. Properties of the 'timed up and go' test: more than meets the eye. *Gerontology*. 2011; **57**(3):203-10. <https://doi.org/10.1159/000314963>

39. Guralnik JM, Simonsick EM, Ferrucci L, et al. A short physical performance battery assessing lower extremity function: association with self-reported disability and prediction of mortality and nursing home admission. *J Gerontol*. 1994; **49**(2):M85-94. <https://doi.org/10.1093/geronj/49.2.m85>

40. Vasunilashorn S, Coppin AK, Patel KV, et al. Use of the Short Physical Performance Battery Score to predict loss of ability to walk 400 meters: analysis from the InCHIANTI study. *J Gerontol A Biol Sci Med Sci*. 2009; **64**(2):223-9. <https://doi.org/10.1093/gerona/gln022>

41. Olsen CF, Bergland A. "Reliability of the Norwegian version of the short physical performance battery in older people with and without dementia". *BMC Geriatr*. 2017; **17**(1):124. <https://doi.org/10.1186/s12877-017-0514-4>

42. Shuman-Paretsky MJ, Belser-Ehrlich J, Holtzer R. Psychometric properties of the Brief Fatigue Inventory in community-dwelling older adults. *Arch Phys Med Rehabil*. 2014; **95**(8):1533-9. <https://doi.org/10.1016/j.apmr.2014.03.026>

43. Fisher MI, Davies C, Lacy H, Doherty D. Oncology Section EDGE Task Force on Cancer: measures of cancer-related fatigue—a systematic review. *Rehabil Oncol*. 2018; **36**(2):93-105. <https://doi.org/10.1097/01.REO.0000000000000124>

44. Karnofsky DA, Burchenal J. The clinical evaluation of chemotherapeutic agents in cancer. In MacLeod CM ed, Evaluation of Chemotherapeutic Agents. New York, NY, USA Columbia University Press, 1949:191-205

45. Péus D, Newcomb N, Hofer S. Appraisal of the Karnofsky Performance Status and proposal of a simple algorithmic system for its evaluation. *BMC Med Inform Decis Mak*. 2013; **13**:72. <https://doi.org/10.1186/1472-6947-13-72>

46. Benedict RHB, Schretlen D, Groninger L, Brandt J. Hopkins Verbal Learning Test–Revised: normative data and analysis of inter-form and test-retest reliability. *Clin Neuropsychol*. 1998; **12**(1):43-55. <https://doi.org/10.1076/clin.12.1.43.1726>

47. Wefel JS, Vardy J, Ahles T, Schagen SB. International cognition and cancer task force recommendations to harmonise studies of cognitive function in patients with cancer. *Lancet Oncol*. 2011; **12**(7):703-8. <https://doi.org/10.1016/s1470-2045(10)70294-1>

48. Tombaugh TN. Trail Making Test A and B: normative data stratified by age and education. *Arch Clin Neuropsychol*. 2004; **19**(2):203-14. <https://doi.org/10.1016/s0887-6177(03)00039-8>

49. Kurita GP, Sandvad M, Lundorff L, De Mattos-Pimenta CA, Højsted J, Sjøgren P. Assessment of cognitive function in patients with metastatic cancer: are we using the right tools? *Palliat Support Care*. 2018; **16**(1):80-9. <https://doi.org/10.1017/s1478951517000694>

50. Ruff RM, Light RH, Parker SB, Levin HS. Benton Controlled Oral Word Association Test: reliability and updated norms. *Arch Clin Neuropsychol*. 1996; **11**(4):329-38. <https://doi.org/10.1093/ARCLIN%2F11.4.329>

51. Hinz A, Mehnert A, Kocalevent RD, et al. Assessment of depression severity with the PHQ-9 in cancer patients and in the general population. *BMC Psychiatry*. 2016; **16**:22. <https://doi.org/10.1186/s12888-016-0728-6>

52. Von Ah D, Jansen CE, Allen DH. Evidence-based interventions for cancer- and treatment-related cognitive impairment. *Clin J Oncol Nurs*. 2014; **18 (Suppl)**:17-25. <https://doi.org/10.1188/14.Cjon.S3.17-25>

53. Marzouk S, Naglie G, Tomlinson G, et al. Impact of androgen deprivation therapy on self-reported cognitive function in men with prostate cancer. *J Urol*. 2018; **200**(2):327-34. <https://doi.org/10.1016/j.juro.2018.02.073>

54. Smith L, Lee JA, Mun J, et al. Levels and patterns of self-reported and objectively-measured free-living physical activity among prostate cancer survivors: a prospective cohort study. *Cancer*. 2019; **125**(5):798-806. <https://doi.org/10.1002/cncr.31857>
